# Driver’s license, head protection devices and severity of motorcyclists’ injuries in traffic accidents

**DOI:** 10.1590/0034-7167-2023-0153

**Published:** 2024-08-26

**Authors:** Albanita Gomes da Costa de Ceballos, Washington José dos Santos, Cristine Vieira do Bonfim

**Affiliations:** I Universidade Federal de Pernambuco, Centro de Ciências Médicas. Recife, Pernambuco, Brazil; II Universidade Federal de Pernambuco, Hospital das Clínicas. Recife, Pernambuco, Brazil; III Fundação Joaquim Nabuco. Recife, Pernambuco, Brazil

**Keywords:** Traffic Accidents, Motorcycle, Head Protective Devices, License, Accidental Injuries, Acidentes de Trânsito, Motocicletas, Dispositivo de Proteção da Cabeça, Habilitação Formal, Lesões Acidentais, Accidentes de Tránsito, Motocicletas, Dispositivos de Protección de la Cabeza, Concesión de Licencias, Lesiones Accidentales

## Abstract

**Objectives:**

to describe traffic accidents involving motorcyclists and analyze the association between possession of a motorcycle driver’s license and use of helmets according to the severity of injuries.

**Methods:**

a cross-sectional study was conducted among all patients hospitalized in the traumatology and orthopedics sector of a public reference hospital in northeastern Brazil.

**Results:**

170 patients were surveyed, the majority were male (95.9%). Their ages ranged from 18 to 67 years. Most were black or brown (52.3%), had completed elementary school (58.9%) and had monthly income smaller than two minimum wages (56.5%). An association was found between being licensed to drive a motorcycle and wearing a helmet. Among those who suffered moderate injuries, this association was OR=5.66(1.85-17.23) and among those who suffered severe injuries it was OR=13.57(2.82-65.14).

**Conclusions:**

people who were licensed to drive motorcycles used a helmet as protective equipment more often and, in accidents, suffered fewer injuries.

## RESUMO

**Objetivos:**

descrever os acidentes de trânsito envolvendo motociclistas e analisar a associação entre ter habilitação para conduzir motocicletas e uso de capacete com a gravidade dos acidentes.

**Métodos:**

estudo transversal com todos os pacientes hospitalizados no setor de traumatologia e ortopedia de um hospital público de referência no Nordeste do Brasil.

**Resultados:**

foram pesquisados 170 pacientes, a maioria do sexo masculino (95,9%). A idade variou entre 18 e 67 anos. Os pesquisados eram de cor preta ou parda (52,3%), tinham estudo fundamental (58,9%) e renda mensal menor que dois salários mínimos (56,5%). Foi encontrada uma associação entre ter habilitação e uso de capacete. Entre os que sofreram acidentes de gravidade moderada, essa associação foi OR=5,66(1,85-17,23) e entre os que sofreram acidentes de gravidade severa foi OR=13,57(2,82-65,14).

**Conclusões:**

pessoas com licença para conduzir motocicletas que usam capacetes como equipamento de proteção, em caso de acidentes, sofrem lesões menos graves.

## RESUMEN

**Objetivos:**

describir los accidentes de tránsito con los motociclistas y evaluar la asociación entre el permiso para conducir motocicletas y el uso del casco con la gravedad de los accidentes.

**Métodos:**

estudio transversal, realizado a los pacientes hospitalizados en el sector de Traumatología y Ortopedia de un hospital público de referencia de la región Nordeste de Brasil.

**Resultados:**

participaron 170 pacientes; la mayoría de ellos del sexo masculino (95,9%), de edad entre 18 y 67 años. Los encuestados eran negros o pardos (52,3%), tenían estudios primarios (58,9%), ingresos mensuales inferiores a dos salarios mínimos (56,5%). Se encontró una asociación entre tener el permiso de conducir y llevar casco. Entre los que sufrieron accidentes moderados, esta asociación fue OR=5,66(1,85-17,23), y entre los que sufrieron accidentes graves fue OR=13,57(2,82-65,14).

**Conclusiones:**

las personas con permiso para conducir motocicletas que utilizaron casco como equipo de protección sufrieron lesiones menos graves en los accidentes.

## INTRODUCTION

Worldwide, traffic accidents involving motorcyclists cause hospitalizations, temporary or permanent disabilities and deaths. It has been estimated that every year, 1.3 million people die as a result of these accidents, and 93% of these deaths occur in low and middle-income countries. In addition, between 20 and 50 million people suffer non-fatal injuries and many of them are left with permanent sequelae^(1)^.

In 2020, 33,716 people died in traffic accidents in Brazil, among whom 11,853 were motorcyclists. Due to the severity of these accidents, about 49% of the motorcyclists involved died on the spot^(2)^. In 2021, 125,882 hospital admissions of motorcyclists injured in traffic accidents were recorded in the Brazilian public healthcare system (*Sistema Único de Saúde*, SUS). The average length of hospitalization was 5.2 days and the total cost was approximately R$ 190 million or 41 million US dollars^(3)^. The state of Pernambuco, where the present study was conducted, ranks second in numbers of deaths due to this cause in Brazil, and 53.6% of the injured individuals die before arriving at the hospital^(2)^.

Approximately 20% of these traffic accident victims report suffering from pain for up to 12 months after the accident^(4)^, which can interfere in performing activities of daily life and can lead to dependence on medications^(5)^, thus affecting mental health and work capacity. The severity of accidents suffered by motorcyclists may be related to risky behaviors that are followed^(6)^, such as not wearing a helmet and driving without a license^(6-8)^.

According to the Brazilian Traffic Code, it is forbidden to ride a motorcycle without using a helmet. Data from the Violence and Accident Surveillance Survey in 23 state capitals and the federal district of Brazil showed that helmet use reduced occurrences of head trauma by 76% and occurrences of referral to another hospital, hospitalization or death by 28%^(9)^. Other studies have estimated that helmet use reduces the risk of fatal accidents by 42% and the risk of head injuries by 69%^(10-11)^.

Possession of a license to drive a motorcycle is also a mandatory item under the Brazilian Traffic Code. Not having this permit can lead to a fine and seizure of the vehicle, in addition to putting the lives of both the motorcyclist and other road users at risk. In a review study^(12)^, it was concluded that mortality due to motorcycle accidents was lower in regions with greater restrictions on obtaining a license to drive this type of vehicle.

Brazilian legislation establishes that the process for obtaining a license to drive motorcycles in Brazil requires, among other things, that candidates need to achieve a pass in a training course that encompasses traffic legislation, defensive driving, notions of first aid, social cooperation in traffic and vehicle operation. Thus, it is believed that individuals who have qualified to drive have become trained to deal with situations with the potential for accident and have a more responsible attitude towards their own safety and that of other people.

Based on the idea that qualified people wear helmets and, in situations of accidents, suffer fewer injuries, the aim of the present study was to describe traffic accidents involving motorcyclists and analyze the association between possession of a license to ride motorcycles and use of helmets, according to the severity of injuries.

## OBJECTIVES

To analyze the association between possession of a license to ride motorcycles and use of helmets, according to the severity of injuries among motorcyclists who suffered accidents.

## METHODS

### Ethical aspects

This research was approved by the Research Ethics Committees of the *Universidade Federal de Pernambuco* and the *Hospital da Restauração Governador Paulo* Guerra (HR). All individuals who agreed to participate in the study signed the Free and Informed Consent Form.

### Design, place of study and period

This study used the EQUATOR (http://www.equator-network.org/) network frame for Observational studies in epidemiology - STROBE.

It is a cross-sectional study carried out between May and September 2016, at the HR in Recife (PE). The RH was chosen for the research because it is the main trauma unit of Pernambuco State and a sentinel unit of surveillance in ground transportation accidents, and has an average of more than 39 thousand hospitalizations per year.

### Population and inclusion and exclusion criteria

There was no sample calculation. The study population consisted of all workers, motorcycle drivers, hospitalized in the Traumatology-Orthopedics ward of the HR. The following inclusion criteria were established: to be employed, to be the driver of the motorcycle at the accident time and to be aged 18 or over. Exclusion criteria were: patients who had altered levels of consciousness that did not respond to the questionnaire, moderate to severe Glasgow coma scores (12 to 3).

### Study protocol

For data collection, a questionnaire elaborated by the researchers was used. This asked about variables relating to sociodemographic characteristics (gender, age and presence of a partner, number of children, schooling, income, number of dependents on this income and race/color), characteristics of the accident (previous accidents, number of previous accidents, nature of the current accident, possession of a license for driving a motorcycle, wearing a helmet at the time of the accident, factors that contributed to the accident and whether the individual felt responsible for the accident) and self-reported injuries according to body area (areas of the body affected and number of injuries).

To make up the variable “severity of the injuries”, we used the sum of the number of areas of the body injured in the accident as a proxy. The accident was considered to be of moderate severity when up to three areas of the body were affected; and was considered to be severe when more than three areas were affected. Cases classified as mild severity were not considered in this study because it was understood that these cases would not be hospitalized in the traumatology sector at the reference hospital where this study was conducted.

The interview time was on average 20 to 30 minutes. The questionnaires were filled out by the researchers who were approaching at the edge of the bed in which the individuals were hospitalized.

### Analysis of results, and statistics

Data were input to and analyzed statistically in the Statistical Package for the Social Sciences (SPSS), version 22.0. The data analysis consisted of descriptive and bivariate stratified analyses in which measurements of associations and their 95% confidence intervals were constructed.

## RESULTS

A total of 170 injured individuals were surveyed. Out of these, 163 (95.9%) were male. The ages of the injured individuals ranged from 18 to 67 years, with a mean of 30.8 years and standard deviation (SD) of 9.8. Living with a partner or partner was reported by 86 people (50.6%) and the mean number of children among the study population was 1.2 (SD = 1.4). A schooling level of incomplete high school or elementary schooling only or no schooling was reported by 100 individuals (58.9%). The income level was less than two minimum monthly wages for 139 individuals (81.8%) and the average number of dependents on this income was 2.4 people (SD = 1.9). Skin color was reported as black or brown by 89 study participants (52.3%).

Regarding previous accidents, 91 drivers (53.5%) reported having suffered such events with motorcycles. The accident that gave rise to hospitalization at the time of this study was caused by some type of collision in 115 cases (67.0%). The participants reported that factors such as holes in the road (potholes) (26; 15.3%) or lack of traffic signs (18; 10.6%) contributed to the occurrence of the accident. At the time of the accident, 38 (22.4%) of the drivers were not wearing helmets and 78 (45.9%) were not qualified to ride motorcycles (i.e. they did not possess a license). When asked about culpability for the accident, 107 (62.9%) of the respondents stated that they did not feel responsible (Table 1).

**Table 1:** Description of traffic accidents with motorcycles

Variable	n	%
Previous motorcycle accident		
Yes	91	53.5
No	79	46.5
Number of previous accidents		
1 or 2	64	70.3
3 or more	27	29.7
Nature of the current accident		
Collision with motor vehicle (other than motorcycle)	72	42.3
Collision with another motorcycle	31	18.2
Collision with pedestrian, bicycle or animal	11	6.5
Falling off	38	22.3
Crash into fixed object	18	10.6
License to drive a motorcycle		
Yes	91	53.8
No	78	46.2
Wearing a helmet at the time of the accident		
Yes	132	77.6
No	38	22.4
Do you feel responsible for the accident?		
Yes, totally	26	15.3
Yes, partially	21	12.4
No	107	62.9
Did not know or did not want to answer	16	9.4

The accidents caused injuries to four or more areas of the body in 76 (44.7%) of those surveyed. The most frequently affected body parts were the head, face or neck, with 105 injuries (17.4%); left leg, ankle or foot, with 79 injuries (13.1%); right forearm, wrist or hand, with 77 injuries (12.3%); and left forearm, wrist or hand with 67 injuries (11.1%). Considering that the individuals surveyed could have suffered injuries in various parts of the body, 603 injuries were counted in total (Table 2).

**Table 2:** Self-reported injuries according to body area among injured motorcyclists

Variable	n	%
Area of the body affected		
Head, face or neck	105	17.41
Chest	18	2.98
Abdomen	13	2.15
Hip or pelvis	17	2.81
Right shoulder or upper arm	28	4.65
Right forearm, wrist or hand	77	12.30
Left shoulder or upper arm	36	5.96
Left forearm, wrist or hand	67	11.10
Right thigh	54	8.95
Right lower leg, ankle or foot	65	10.76
Left thigh	44	7.30
Left lower leg, ankle or foot	79	13.10
Number of injuries resulting from the accident		
1 to 3	94	55.3
4 or more	76	44.7

*Each individual surveyed could have suffered injuries in different areas of the body; The total number of injuries according to the area of the body reached 603.*

In the group of patients who suffered an accident that was considered moderate, non-qualified individuals (i.e. without a driver’s license) were 5.66 times more likely not to wear a helmet than were qualified individuals, while in the group of patients who suffered an accident that was considered severe, this chance was 13.57 (Table 3).

**Table 3:** Stratified analysis on the association between possession of a license and helmet use, according to injuries caused by the accident

Severity of the accident	Possession of license	Helmet use						
		Yes		No		OR	95% CI	
		n	%	n	%		Lower	Upper
Moderate (up to 3 injuries)								
	Yes	46	63.9	5	23.8	-	-	-
	No	26	36.1	16	76.2	5.66	1.85	17.23
	Total	72	100	21	100			
Severe (more than 3 injuries)								
	Yes	38	64.4	2	11.8	-	-	-
	No	21	35.6	15	88.2	13.57	2.82	65.14
	Total	59	100	17	100			

## DISCUSSION

Most of the individuals surveyed in the present study were young men, and this profile was the same as found in the literature^(13)^. This emphasizes the idea that motorcycles are a means of transportation that forms a cheap option for rapid locomotion. While young people make up about 10% of the driver population, their mortality rate in motorcycle accidents is up to three times higher^(14)^. A survey of Taiwanese motorcyclists showed that novice drivers who were underage or unlicensed were more likely to have fatal accidents. Male gender, helmet wearing and front impacts play significant roles in accidents among novice motorcyclists^(15)^.

Lower accident rates have been reported in areas with stricter regulations for obtaining a driver’s license^(16)^. However, in Brazil, the high costs involved and time required for acquiring the national driver’s license (CNH) contribute to the data on the lack of a driver’s license among motorcyclists^(16)^.

Despite the prohibition of driving a motorcycle without a helmet, more than 20% of the motorcyclists in the present survey were violating this traffic rule at the time of their accident, and thus were accepting the risk of head and neck injuries and death. Previous studies have shown that more than 40% of deaths in traffic are due to traumatic brain injuries^(17)^ and that the use of a helmet protects against death and head injuries, with significant odds ratios of 0.58 and 0.31, respectively^(10)^.

Brazilian legislation recommends the use of full helmets, covering the entire head. A review of the literature that evaluated types of helmets and the risk of head and neck injury concluded that fully closed helmets reduced head and neck injuries in motorcycle accidents to a greater extent than other types of helmet^(18)^.

The number of injuries resulting from accidents that was found in the present study was similar to the findings from another study in Brazil that showed that 45% of the patients were polytraumatized^(19)^. Moreover, with regard to recurrence of motorcycle accidents, other studies showed that most of the interviewees had suffered previous accidents^(20)^ and that this reality was even more common among delivery motorcyclists or motorcycle couriers^(21)^, thus demonstrating the poor working conditions and risks suffered by these professionals.

In addition to the head region, the body parts most affected among the motorcyclists who participated in this study were the regions of the forearm, wrist and hand on both sides, as well as the legs, ankles and feet. In a study on motorcyclists treated at Hospital das Clínicas, University of São Paulo, the lower limbs were the region most affected, followed by the upper limbs^(20)^. Although injuries in these areas of the body are less often fatal than are head injuries, they often require surgery and use of orthotics or prostheses, and can also give rise to temporary or permanent incapacity for work, thus burdening the healthcare and social security services.

The results from the present study show that, for both moderate and severe injuries, possessing a license to ride motorcycles was associated with use of a helmet while driving the motorcycle. Thus, the motorcyclists surveyed here who had a license seemed to be taking a more responsible attitude towards driving their vehicle, through using the mandatory item (helmet) on the day of the accident and, consequent to the accident, suffered a lower number of injuries.

Another finding from this study was the participants’ sense of responsibility for the accident. It was seen that although many of these motorcyclists did not have a driver’s license and were not wearing helmets at the time of the accident, thus violating the traffic laws, more than 60% of the interviewees did not feel guilty about the accident.

### Study limitations

This research may have limitations related to some omission of information due to fear of judgment or legal punishment. Such as, for example, in question about license to drive a motorcycle, since it is prohibited in Brazil. Another limitation would be the lack of adoption of a trauma index to classify the severity of injuries, given the absence of clinical variables in the data collected.

### Contributions to the fields of Nursing, Health or Public Policy

Nursing professionals participate in this from rescuing the motorcycle accident victim to hospital care and post-hospital care. Understanding the relationship between risk behaviors and injury severity among motorcyclists points to the need to reinforce prevention, surveillance and inspection strategies. As professionals working at all levels of health care, in schools, universities, health management, rehabilitation centers, industrier, companies and many others spaces, they can discuss and develop health education strategies that reduce the number and the severity of accidents.

## CONCLUSIONS

The number of motorcyclists who did not have a driver’s license and yet were driving a motorcycle shows a situation that requires attention. Moreover, an association between possession of a license to ride motorcycles and helmet use was demonstrated, and this was stronger among the victims of severe accidents.

Therefore, is important to establish educational programs about traffic accidents involving nurses and other health professionals working in emergence care. In another hand, is important discuss more severe legal punishments to drivers who adopt risky traffic behaviors in order to reduce the high costs of hospitalization and lost lives among this population.
